# Pathophysiology of LV Remodeling Following STEMI: A Longitudinal Diffusion Tensor CMR Study

**DOI:** 10.1016/j.jcmg.2022.04.002

**Published:** 2023-02

**Authors:** Arka Das, Christopher Kelly, Irvin Teh, Christian T. Stoeck, Sebastian Kozerke, Noor Sharrack, Peter P. Swoboda, John P. Greenwood, Jürgen E. Schneider, Sven Plein, Erica Dall’Armellina

**Affiliations:** a Department of Biomedical Imaging Science, Leeds Institute of Cardiovascular and Metabolic Medicine, University of Leeds, Leeds Teaching Hospitals NHS Trust, Leeds, United Kingdom; b Institute for Biomedical Engineering, University and ETH Zurich, Zurich, Switzerland

**Keywords:** adverse remodeling, cardiac magnetic resonance (CMR), diffusion tensor (DT) imaging, heart failure, myocardial infarction (MI), CMR, cardiac magnetic resonance, DT, diffusion tensor, E2A, secondary eigenvector angle, IMH, intramyocardial hemorrhage, LGE, late gadolinium enhancement, LHM, myocytes with left-handed orientation, LVEDVi, left ventricular end-diastolic volume indexed for body surface area, LVEF, left ventricular ejection fraction, MI, myocardial infarction, MVO, microvascular obstruction, PCI, percutaneous coronary intervention, RHM, myocytes with right-handed orientation, STEMI, ST-segment elevation myocardial infarction

## Abstract

**Background:**

Adverse LV remodeling post–ST-segment elevation myocardial infarction (STEMI) is associated with a poor prognosis, but the underlying mechanisms are not fully understood. Diffusion tensor (DT)-cardiac magnetic resonance (CMR) allows in vivo characterization of myocardial architecture and provides unique mechanistic insight into pathophysiologic changes following myocardial infarction.

**Objectives:**

This study evaluated the potential associations between DT-CMR performed soon after STEMI and long-term adverse left ventricular (LV) remodeling following STEMI.

**Methods:**

A total of 100 patients with STEMI underwent CMR at 5 days and 12 months post-reperfusion. The protocol included DT-CMR for assessing fractional anisotropy (FA), secondary eigenvector angle (E2A) and helix angle (HA), cine imaging for assessing LV volumes, and late gadolinium enhancement for calculating infarct and microvascular obstruction size. Adverse remodeling was defined as a 20% increase in LV end-diastolic volume at 12 months.

**Results:**

A total of 32 patients experienced adverse remodeling at 12 months. Compared with patients without adverse remodeling, they had lower FA (0.23 ± 0.03 vs 0.27 ± 0.04; *P <* 0.001), lower E2A (37 ± 6° vs 51 ± 7°; *P <* 0.001), and, on HA maps, a lower proportion of myocytes with right-handed orientation (RHM) (8% ± 5% vs 17% ± 9%; *P <* 0.001) in their acutely infarcted myocardium. On multivariable logistic regression analysis, infarct FA (odds ratio [OR]: <0.01; *P =* 0.014) and E2A (OR: 0.77; *P =* 0.001) were independent predictors of adverse LV remodeling after adjusting for left ventricular ejection fraction (LVEF) and infarct size. There were no significant changes in infarct FA, E2A, or RHM between the 2 scans.

**Conclusions:**

Extensive cardiomyocyte disorganization (evidenced by low FA), acute loss of sheetlet angularity (evidenced by low E2A), and a greater loss of organization among cardiomyocytes with RHM, corresponding to the subendocardium, can be detected within 5 days post-STEMI. These changes persist post-injury, and low FA and E2A are independently associated with long-term adverse remodeling.

## Background

Following acute myocardial infarction (MI), reduced contractility in the left ventricle leads to an acute increase in loading conditions and triggers adaptive neurohormonal mechanisms.^1^ Failure to normalize the increased wall stress results in progressive cavity dilatation and reduction in contractility, a process known as adverse remodeling, which is associated with reduced survival.^2^ The exact mechanisms underpinning adverse remodeling are incompletely understood. Cardiac magnetic resonance (CMR) offers a range of techniques for quantifying edema, scarring, impairment in myocardial deformation, and contractility post-MI. The emergence of diffusion tensor (DT)-CMR now also permits the assessment of the organization and integrity of underlying microstructural components in vivo.^3^

Reorientation of laminar “sheetlets” through the cardiac cycle correlates with myocardial strain and drives LV wall thickening in systole. In DT-CMR, the absolute secondary eigenvector angle (E2A) is a measure of the angularity of myocardial sheetlets. Global reduction in E2A during systole has been used to explain mechanistic deficiencies in wall strain and predict LV remodeling in patients with dilated cardiomyopathy.^3^ In patients with MI, regional reduction in E2A in acutely infarcted segments has been shown to correlate with lower left ventricular ejection fraction (LVEF) at 3 months.^4^ DT CMR can also characterize the helical arrangement of cardiomyocytes in vivo, as demonstrated on dissection plates.^5^ In infarct segments, reductions in the proportion of myocytes with right-handed orientation (RHM) have been described, attributed to the loss of organization among subendocardial myocytes that is associated with lower LVEF post-MI.^4^ DT-CMR can also infer tissue characteristics by measuring the mean diffusivity (MD) and fractional anisotropy (FA) of diffusion within the myocardium. MD can detect the presence of edema with higher signal contrast than T1 and T2 mapping,^6^ as well as depicting areas of interstitial fibrosis.^7^^,^^8^ Reduced FA can signify collagen infiltration and cardiomyocyte disorganization,^9^^,^^10^ and it has been shown to be independently predictive of long-term LVEF post-MI.^4^ Hence DT-CMR can provide mechanistic insights into the pathophysiologic mechanisms that drive adverse LV remodeling post-MI.

We therefore sought to: 1) explore baseline and long-term changes in DT-CMR parameters over 12 months post–ST-segment elevation myocardial infarction (STEMI); and 2) assess the potential association between early DT-CMR measures and long-term adverse remodeling.

## Methods

### Patient recruitment

Patients with a first STEMI were prospectively recruited from a single tertiary center (The Leeds Teaching Hospitals NHS Trust, Leeds, United Kingdom) between 2019 and 2020. Study inclusion criteria were: 1) acute STEMI as defined by current international guidelines;^11^ 2) revascularization by primary percutaneous coronary intervention (PCI) within 12 hours after onset of symptoms; and 3) no contraindications to CMR. Exclusion criteria were: 1) previous revascularization procedure (coronary artery bypass grafting or PCI); 2) known cardiomyopathy; 3) severe valvular heart disease; 4) atrial fibrillation; and 5) hemodynamic instability lasting longer than 24 hours following PCI. The study protocol was approved by The Institutional Research Ethics Committee (NIHR 33963, REC 17/YH/0062) and complied with the Declaration of Helsinki; all patients gave written informed consent for their participation.

### Image acquisition

The study protocol included a CMR scan within 3 to 7 days of index presentation (early scan) and follow-up imaging at 12 months. CMR examinations were performed on a 3.0-T system (Philips Achieva). The CMR protocol included the following: full LV coverage by functional cine and late gadolinium enhancement (LGE) imaging, 3 anatomically matching short-axis slices (located at the base, mid, and apex) by DT-CMR, modified Look-Locker inversion (5[3]3 MOLLI) T1 mapping, T2∗ mapping, and postcontrast T1 mapping (see the Supplemental Methods for pulse sequence parameters).

DT-CMR data were acquired using electrocardiogram-gated second-order motion-compensated single-shot spin echo (SE) planar imaging sequence with asymmetrical bipolar diffusion waveforms and respiratory navigator tracking (TE/TR, 89 ms/3 RR intervals; flip angle, 90°; field of view, 238 × 238 mm; matrix size, 108 × 105; acquired in-plane resolution, 2.20 × 2.27; slice gap, 8 mm; reconstructed voxel size, 1.7 × 1.7 × 8 mm; sensitivity encoding [SENSE] acceleration, 1.8). Each DT-CMR data set constituted 18 non-collinear diffusion-weighted acquisitions with b-values of 100 s/mm^2^ (×3), 200 s/mm^2^ (×3), and 500 s/mm^2^ (×12). On the basis of cine data, trigger delay was set individually for each patient to coincide with 60% peak systole, and the center of k-space was at 85% of peak systole.

### Image analysis

Cine, mapping, and LGE data were analyzed using cvi42 software (Circle Cardiovascular Imaging Inc) to derive LV volumes, LVEF, and tissue characteristics, including native T1, T2∗, extracellular volume (ECV), infarct size, and microvascular obstruction (MVO), as previously described^4^ (see the Supplemental Methods for further details). On T2∗ maps, an area of reduced signal intensity within infarcted myocardium with T2∗ <20 ms was considered to confirm the presence of intramyocardial hemorrhage (IMH).^12^ Adverse remodeling was defined as an increase in left ventricular end-diastolic volume indexed for body surface area (LVEDVi) >20% at 12 months from baseline.^13^

DT-CMR data processing was performed using in-house–developed MATLAB software, as described previously.^4^ Quality control was undertaken by visual assessment; diffusion-weighted images corrupted by artifact or failed registration were omitted from further processing. Tensor eigenvalues, MD, FA, helix angle (HA), and E2A maps were calculated on the basis of the tensors derived from diffusion-weighted imaging data acquired at diffusion gradients with b = 100, 200, and 500 s/mm^2^ images. Endocardial and epicardial borders were manually delineated on the basis of the reconstructed nondiffusion-weighted data; cine images in the same phase of the cardiac cycle were used as a visual reference for more precise recognition of borders. Both region of interest (ROI)–based analysis and segmental analysis were performed as described in the following subsections.

#### ROI analysis

ROIs manually drawn in accordance with standards set by the European Association for Cardiovascular Imaging^14^ were used for the analysis of T1, ECV, MD, and FA. For each patient in each affected slice, 3 ROIs were drawn, corresponding to infarct (positive for LGE), adjacent edematous myocardium (negative LGE, raised native T1 [departmental threshold >1,240 ms]), and remote myocardium (opposite the infarct). To avoid the paramagnetic susceptibility effects of iron, care was taken to avoid sampling ROIs from areas of MVO (as seen on LGE) and IMH (identified using T2∗ mapping). ROIs were drawn and copied across to parametric and DT-CMR maps. Infarct ROIs on parametric and DT-CMR maps were adjusted so they did not sample areas of IMH. For each patient, the location of ROIs from the early scan were used as a visual reference for sampling ROIs from 12-month scans, so that sampling occurred from nearly identical locations. Examples of ROI sampling are shown in Supplemental Figures 1 and 2.

#### Segmental analysis

Because HA and E2A values are expected to vary transmurally in healthy persons, segmental analysis was preferred over the ROI approach. HA maps were described by classifying voxels to 1 of 3 groups—myocytes with left-handed orientation (LHM) (−90° ≤ HA <−30°), myocytes with circumferential orientation (−30° ≤ HA ≤30°) and RHM (30° < HA ≤90°)—and quantitative markers derived as the respective myocardial proportions of each type, as done previously.^4^ After dividing each slice into 6 equiangular segments starting from the anterior interventricular junction,^15^ segmental E2A and HA averages were derived. Segments were classified as infarct (visual evidence of LGE with enhancement of >10% of pixels), adjacent (located on the same plane contiguously to infarct segments), and remote (opposite the infarct), and they were then averaged to provide 1 data point for each zone per patient. Intraobserver reproducibility for the analysis of DT-CMR data using the methods mentioned earlier was demonstrated previously.^4^ Intrasubject reproducibility is provided in the Supplemental Methods (Supplemental Table 1, Supplemental Figure 3).

### Statistical analysis

Statistical analyses were performed using SPSS software version 21.0 (IBM Corp). Normality was checked using the Shapiro-Wilk test. The primary end point of this study was the occurrence of adverse LV remodeling, defined by a 20% increase in LVEDVi at 12 months from baseline.^13^ Secondary end points comprised serial changes in LVEDVi and LVEF over 12 months. Continuous variables are expressed as mean ± SD or median (IQR), as appropriate. Comparison among quantitative variables was performed by independent-sample parametric (unpaired Student’s *t*-test) or nonparametric (Mann-Whitney) statistical tests, as appropriate. For repeated measurements, paired Student's *t*-tests and analysis of variance with Bonferroni post hoc comparisons were used. Categorical data were compared using Pearson chi-square tests. Correlations between DT-CMR or parametric mapping values from remote, adjacent, and infarct regions and LV volumes were assessed by Pearson correlation analysis. Univariate analyses were performed to identify predictors of LVEF and LVEDVi at 12 months. Possible collinearity among candidate predictors was assessed using variance-inflation factors with threshold equal to 5. To avoid overfitting, only variables with a value of *P* < 0.05 in the univariate analysis were included in a multivariable linear regression analysis. Binary logistic regression models were used to identify associates of adverse remodeling at 12 months and, alongside DT-CMR parameters, included only the best CMR covariates (acute LVEF and infarct size) to reduce the number of analyzable parameters with respect to our sample size and improve the statistical robustness of the model. Statistical significance of the differences between receiver-operating characteristic curves was assessed using the method of DeLong et al,^16^ and the optimal thresholds were determined on the basis of the maximum Youden Index. All tests were assumed to be statistically significant when *P <* 0.05.

## Results

### Patient characteristics

**Figure 1:**
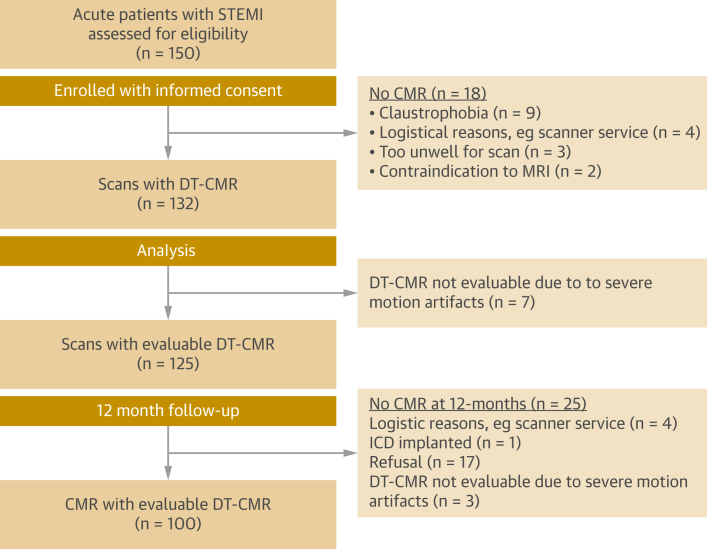
Flowchart of Study Enrollment From the 150 enrolled patients, 100 completed early and 12-month scans. CMR = cardiac magnetic resonance; DT = diffusion tensor; ICD = implantable cardioverter-defibrillator; MRI = magnetic resonance imaging; STEMI = ST-segment elevation myocardial infarction.

The Central Illustration outlines the main outcomes from this study. Figure 1 shows the study flowchart. Of the 150 enrolled patients, 100 (male-to-female ratio: 80:20, aged 59 ± 10 years) completed early (5 ± 2 days) and 12-month scans (391 ± 73 days); 43% of the patients presented with anterior STEMI (Table 1).

**Central Illustration:**
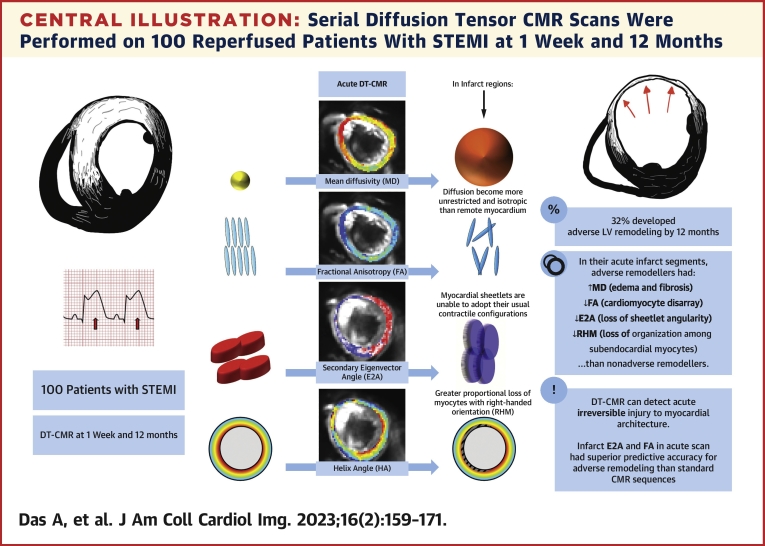
Serial Diffusion Tensor CMR Scans Were Performed on 100 Reperfused Patients With STEMI at 1 Week and 12 Months Acutely infarcted myocardium had higher mean diffusivity (MD) and lower fractional anisotropy (FA), suggesting that diffusion becomes more unrestricted and isotropic from edema and cardiomyocyte disorganization than remote myocardium. The myocardial sheetlets are unable to adopt their usual orientation in systole, as suggested by low absolute secondary eigenvector angle (E2A) values, and they have fewer myocytes with right-handed orientation (RHM) on helix angle maps, thus highlighting the early loss of organization among subendocardial myocytes. Acute infarct fractional anisotropy and secondary eigenvector angle were independently associated with adverse left ventricular (LV) remodeling at 12 months, with better accuracy than other cardiac magnetic resonance (CMR)–derived biomarkers. DT = diffusion tensor; STEMI = ST-segment elevation myocardial infarction.

**Table 1:** Baseline Demographics, Presenting Characteristics, and Post-MI Pharmacotherapy

	All Patients (n = 100)	No Adverse Remodeling at 12 mo (n = 68)	Adverse Remodeling at 12 mo (n = 32)	*P* Value
Baseline demographics				
Age, y	59 ± 10	59 ± 10	60 ± 11	0.72
Male	80 (80)	54 (79)	26 (81)	0.83
BSA, m^2^	1.94 ± 0.17	1.93 ± 0.18	1.95 ± 0.17	0.67
Hypertension	25 (25)	17 (25)	8 (25)	1.00
Diabetes mellitus	18 (18)	13 (19)	5 (16)	0.67
Current smoker	27 (27)	19 (27)	8 (25)	0.76
Heart rate, beats/min	77 ± 14	76 ± 13	79 ± 14	0.88
Systolic blood pressure, mm Hg	133 ± 53	132 ± 48	135 ± 44	0.76
Diastolic blood pressure, mm Hg	75 ± 12	74 ± 10	76 ± 8	0.92
Presenting characteristics				
Pain to balloon time, min	245 ± 170	265 ± 202	213 ± 94	0.25
Culprit artery				
Left main stem	0 (0)	0 (0)	0 (0)	1.00
Left anterior descending	43 (43)	27 (40)	16 (50)	0.33
Left circumflex	16 (16)	10 (15)	6 (19)	0.61
Right coronary	41 (41)	31 (45)	10 (31)	0.17
TIMI coronary flow grade pre-PPCI				
<3	99 (99)	67 (99)	32 (100)	0.68
3	1 (1)	1 (1)	0 (0)	0.49
TIMI coronary flow grade post-PPCI				
<3	3 (3)	0 (0)	3 (9)	0.01
3	97 (97)	68 (100)	29 (91)	0.06
Pharmacologic therapy post-MI				
Aspirin	100 (100)	68 (100)	32 (100)	1.00
Adenosine diphosphate receptor antagonist	100 (100)	68 (100)	32 (100)	1.00
ACE inhibitor or angiotensin II receptor blocker	100 (100)	68 (100)	32 (100)	1.00
Beta-blocker	97 (97)	66 (97)	31 (97)	0.96

Values are mean ± SD or n (%).

ACE = angiotensin-converting enzyme; BSA = body surface area, MI = myocardial infarction; PPCI = primary percutaneous coronary intervention; TIMI = Thrombolysis In Myocardial Infarction.

### DT-CMR acquisition

**Figure 2:**
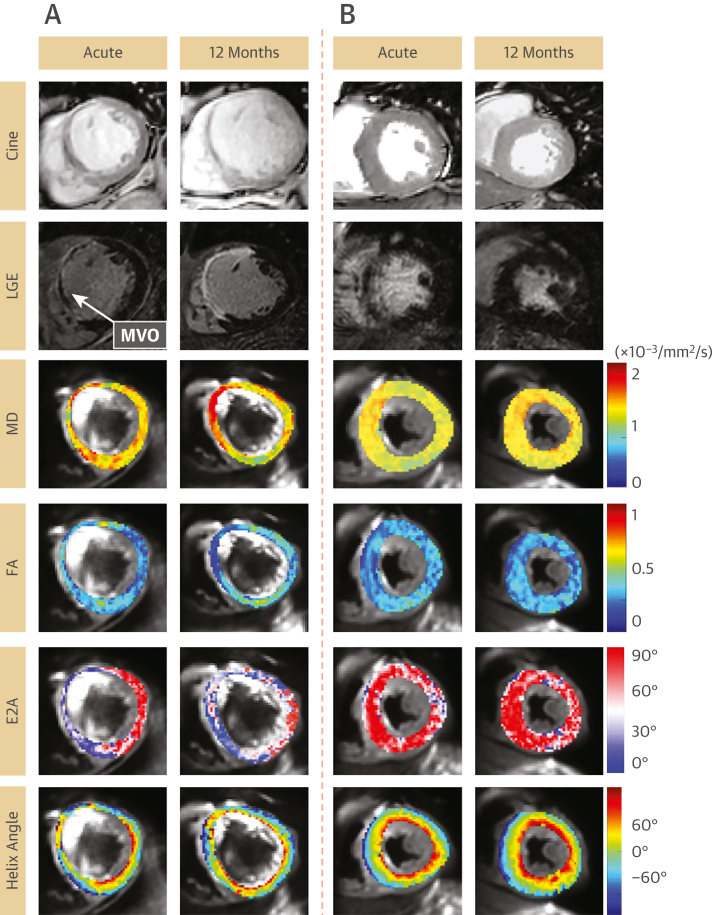
Representative CMR Images of 2 Separate Patients **(A)** Short-axis images obtained from the early and 12-month scans of a 70-year-old man who initially presented with anterior ST-segment elevation and underwent primary PCI to his left anterior descending artery. His acute LVEF remained severely impaired (30%) at 12 months, but his LVEDV increased from 172 mL to 228 mL, thus fulfilling the criteria for adverse remodeling. In the early scan, LGE demonstrated transmural infarction of basal anterior and septal walls. Corresponding areas on diffusion tensor maps show high MD, low FA, low E2A, and reduction of RHM **(red and orange pixels)**. By 12 months, the anterior and septal walls have thinned and remain transmurally infarcted. MD was increased in areas corresponding to scar, which in the absence of edema is suggestive of expansion in ECV. FA remained low, indicating underlying collagen deposition and cardiomyocyte disorganization. The E2A remained low, suggesting the myocardial sheetlets were unable to adopt their usual systolic configuration, and the relative absence of RHM suggests loss of organization among subendocardial myocytes. **(B)** Short-axis images of a 65-year-old man who also presented with anterior ST-segment elevation and underwent primary PCI to the left anterior descending artery. By 12 months, LVEF improved from 44% to 56%, whereas LVEDV remained unchanged at 115 mL. In the early scan, LGE demonstrated subendocardial infarction of the septal walls. MD was raised acutely in infarcted areas, but not as high as in the patient in **A.** FA, E2A, and proportions of RHM in infarct areas appeared relatively preserved (higher) compared with the patient in **A,** and they did not change over 12 months, thus demonstrating that the preservation of sheetlet angularity (higher E2A) and organization among subendocardial myocytes (higher RHM) were detectable in the early scan. E2A = secondary eigenvector angle; ECV = extracellular volume; FA = fractional anisotropy; LGE = late gadolinium enhancement; LVEDV = left ventricular end-diastolic volume; LVEF = left ventricular ejection fraction; MD = mean diffusivity; MVO = microvascular obstruction; PCI = percutaneous coronary intervention; RHM = myocytes with right-handed orientation; other abbreviation as in Figure 1.

The mean acquisition time for DT-CMR was 13 ± 5 minutes. Representative images are shown in Figure 2. In all patients, apical DT-CMR slices were not used for the final analysis because of frequent artifacts from unsuppressed fat, signal loss, and suboptimal signal-to-noise ratio.

### Early and 12-month scan results

Results from the early scans are shown in Table 2. The mean infarct size was 22% ± 14% of LV mass. MD was higher in infarct regions than in remote regions (1.73 ± 0.12 vs 1.47 ± 0.08 × 10^-3^ mm^2^/s; *P <* 0.001). FA, absolute E2A, and the proportion of RHM were all lower in infarcted than remote myocardium (FA, 0.26 ± 0.04 vs 0.36 ± 0.04; *P <* 0.001; E2A, 46 ± 9° vs 52 ± 9°; *P <* 0.001; RHM, 14% ± 8% vs 24% ± 11%; *P <* 0.001). Subgroup analysis of patients with and without MVO are provided in the Supplemental Methods (including Supplemental Tables 2 and 3).

**Table 2:** Results From the Early CMR Scan

	Early Scan			
	All Patients (n = 100)	No Adverse Remodeling at 12 mo (n = 68)	Adverse Remodeling at 12 mo (n = 32)	*P* Value
LVEF, %	43 ± 9	46 ± 8	37 ± 9	<0.001
BSA-indexed LV end-diastolic volume, mL/m^2^	79 ± 15	78 ± 13	82 ± 19	0.950^a^
LV mass, g	112 ± 28	108 ± 24	121 ± 33	0.031
Infarct size, % of LV mass	22 ± 14	12 ± 10	27 ± 18	<0.001
Microvascular obstruction size, g	1.4 ± 2.8	0.5 ± 1.6	3.1 ± 4.1	<0.001^a^
Wall thickness of infarct segment, mm	7.8 ± 1.4	7.8 ± 1.4	7.7 ± 1.3	0.613
Remote regions				
Native T1, ms	1,196 ± 6 0	1,186 ± 62	1,217 ± 50	0.015
ECV, %	27 ± 5	27 ± 5	27 ± 3	0.681
MD, × 10^-3^ mm^2^/s	1.47 ± 0.08	1.45 ± 0.08	1.52 ± 0.09	<0.001
FA	0.36 ± 0.04	0.36 ± 0.04	0.35 ± 0.03	0.362^a^
Absolute E2A, °^b^	52 ± 9	52 ± 9	52 ± 10	0.975
RHM, %^b^	24 ± 11	23 ± 10	26 ± 11	0.264^a^
Circumferential myocyte orientation, %^b^	64 ± 14	65 ± 13	60 ± 15	0.181^a^
LHM, %^b^	13 ± 7	12 ± 6	14 ± 8	0.719^a^
Adjacent regions				
Native T1, ms	1,295 ± 71	1,299 ± 54	1,315 ± 48	0.096
ECV, %	31 ± 6	32 ± 6	29 ± 4	0.079
MD, × 10^-3^ mm^2^/s	1.60 ± 0.10	1.58 ± 10	1.63 ± 0.09	0.088
FA	0.33 ± 0.04	0.33 ± 0.04	0.32 ± 0.03	0.836
Absolute E2A, °^b^	50 ± 8	51 ± 8	48 ± 6	0.078
RHM, %^b^	15 ± 7	15 ± 7	14 ± 6	0.447
Circumferential myocyte orientation, %^b^	71 ± 11	72 ± 11	71 ± 10	0.674
LHM, %^b^	14 ± 6	13 ± 7	15 ± 6	0.234
Infarct regions				
Native T1, ms	1,488 ± 111	1,471 ± 113	1,522 ± 102	0.052
ECV, %	54 ± 11	53 ± 11	57 ± 10	0.081
Presence of IMH on T2∗ mapping	34 (34)	16 (24)	18 (56)	<0.001
MD, × 10^-3^ mm^2^/s	1.73 ± 0.12	1.71 ± 0.12	1.78 ± 0.13	0.053
FA	0.26 ± 0.04	0.27 ± 0.04	0.23 ± 0.03	<0.001
Absolute E2A, °^b^	46 ± 9	51 ± 7	37 ± 6	<0.001
HA transmural gradient, °/%	−0.35 ± 0.28	−0.31 ± 0.10	−0.38 ± −0.32	0.063
RHM, %^b^	14 ± 8	17 ± 9	8 ± 5	<0.001^a^
Circumferential myocyte orientation, %^b^	68 ± 8	67 ± 13	72 ± 12	0.722
LHM, %^b^	18 ± 10	16 ± 9	20 ± 9	0.168

Values are mean ± SD or n (%).

CMR = cardiac magnetic resonance; E2A = secondary eigenvector angle; ECV = extracellular volume; FA = fractional anisotropy; HA = helix angle; IMH = intramyocardial hemorrhage; LHM = myocytes with left-handed orientation; LV = left ventricular; LVEF = left ventricular ejection fraction; MD = mean diffusivity; RHM = myocytes with right-handed orientation; other abbreviation as in Table 1.

a Mann-Whitney analysis for nonparametric data.

b Derived using segmental analysis. Departmental 3.0-T scanner reference range for native T1 is 1,190 ± 50 ms.

By the 12-month scans, the mean LVEDVi increased from 79 ± 15 mL/m^2^ to 85 ± 22 mL/m^2^ *(P =* 0.015), whereas the mean LVEF improved from 43% ± 9% to 49% ± 9% (*P <* 0.001). In chronic infarct zones, MD remained higher than remote (1.70 ± 0.14 vs 1.48 ± 0.06 × 10^-3^ mm^2^/s; *P <* 0.001), whereas FA, absolute E2A, and proportions of RHM were lower in infarcted than remote myocardium (FA, 0.26 ± 0.04 vs 0.34 ± 0.03; *P <* 0.001; E2A, 44 ± 10 vs 48 ± 7; *P <* 0.01; RHM, 14 ± 8 vs 21 ± 8; *P <* 0.001) (Supplemental Table 4).

### Comparison of adverse vs nonadverse remodelers

**Figure 3:**
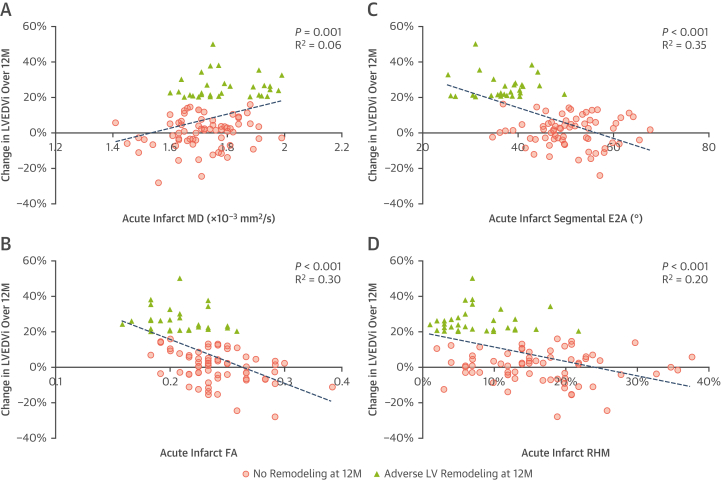
Correlations Between Early DT-CMR Parameters and LV Size at 12 Months **(A)** Increased MD, **(B)** decreased FA, **(C)** decreased absolute E2A, and **(D)** decreased proportions of RHM in acutely infarcted myocardium were associated with the degree of serial increase in body surface area (BSA)–indexed left ventricular end-diastolic volume (LVEDVi) over 12 months following STEMI. LV = left ventricular; other abbreviations as in Figures 1 and 2.

**Figure 4:**
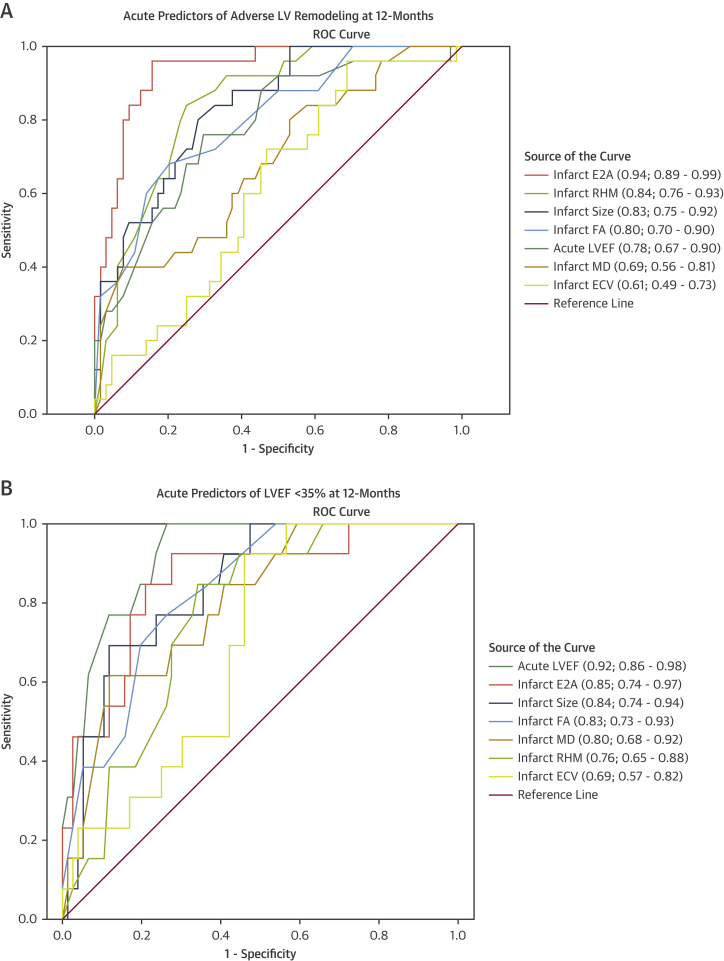
ROC Curves for Prediction of Adverse LV Remodeling and LVEF <35% at 12 Months Comparison of receiver-operating characteristic (ROC) curves with 95% CIs of diffusion tensor CMR parameters, acute LVEF, acute infarct size, and infarct ECV. The absolute E2A had the highest area under the curve for predicting adverse remodeling. Abbreviations as in Figures 1 to 3.

Of the 100 patients, 32 developed adverse remodeling by their 12-month scan. These patients had lower FA (0.23 ± 0.03 vs 0.27 ± 0.04; *P <* 0.001), absolute E2A (37 ± 6° vs 51 ± 7°; *P <* 0.001), and proportion of RHM (8% ± 5% vs 17% ± 9%; *P <* 0.001) in their acutely infarcted myocardium than nonadverse remodelers (Table 2). Univariate linear regression analysis identified several CMR-based characteristics from the early scan to be significantly associated with serial change in LVEDVi at 12 months (Table 3). Acute infarct FA (r^2^ = 0.305), E2A (r^2^ = 0.353), and RHM (r^2^ = 0.200) all correlated with change in LVEDVi over 12 months (Figure 3). Following multivariate regression analysis adjusting for factors including baseline LVEF, LVEDVi, infarct size, extent of MVO, and infarct ECV, only infarct FA (standardized β −0.364; *P =* 0.001) and infarct E2A (standardized β −0.336; *P =* 0.001) were independently associated with change in LVEDVi over 12 months. On multivariable logistic regression analysis, FA (odds ratio [OR]: <0.01; *P =* 0.014) and absolute E2A (OR: 0.77; *P =* 0.001) of infarcted myocardium were the only significant factors independently associated with adverse LV remodeling at 12 months after adjusting for LVEF and infarct size (Table 4). ROC analysis for the prediction of adverse remodeling (ie, increase in LVEDV >20% over 12 months) demonstrated acute infarct E2A and RHM to have higher areas under the curve than LGE and acute LVEF (Figure 4A). Acute infarct E2A of <45° had 84% sensitivity and 85% specificity, and RHM <12% had 85% sensitivity and 77% specificity for predicting adverse remodeling.

**Table 3:** Early Predictors of 12-Month LVEDVi and LVEF Using Univariate and Multivariate Regression

CMR Findings at Baseline	Correlation With Change in LVEDVi at 12 mo				Correlation With LVEF at 12 mo			
	Univariate Analysis		Multivariate Analysis		Univariate Analysis		Multivariate Analysis	
	r^2^	*P* Value	Beta	*P* Value	r^2^	*P* Value	Beta	*P* Value
LVEF, %	0.247	<0.001	−0.023	0.849	0.486	<0.001	0.352	0.001
BSA-indexed LV end-diastolic volume, mL/m^2^	0.076	0.005	0.141	0.144	0.225	<0.001	−0.212	0.014
Infarct size, % of LV mass	0.168	<0.001	−0.009	0.937	0.226	<0.001	−0.040	0.682
MVO size, g	0.163	<0.001	0.111	0.270	0.172	<0.001	−0.040	0.655
Infarct regions								
ECV, %	0.073	0.008	−0.071	0.445	0.181	<0.001	−0.019	0.814
MD, × 10^-3^ mm^2^/s	0.033	0.001	−0.024	0.809	0.091	0.003	0.025	0.772
FA	0.305	<0.001	−0.364	0.001	0.244	<0.001	0.246	0.008
Absolute E2A, °^a^	0.353	<0.001	−0.336	0.001	0.151	<0.001	0.016	0.853
RHM, %^a^	0.200	<0.001	−0.132	0.185	0.233	<0.001	0.177	0.049

LVEDVi = left ventricular end-diastolic volume indexed for body surface area; MVO = microvascular obstruction; other abbreviations as in Tables 1 and 2.

a Derived using segmental analysis.

**Table 4:** Multivariate Logistic Regression Analysis of Early Predictors of Adverse LV Remodeling

	Odds Ratio	95% CI		*P* Value
		Lower	Upper	
LVEF, %	0.98	0.87	1.09	0.658
Infarct size, % of LV mass	1.04	0.96	1.14	0.326
MVO, g	1.08	0.79	1.50	0.627
Infarct FA	<0.01	<0.01	0.01	0.014
Infarct E2A, °	0.77	0.66	0.89	0.001
Infarct RHM, %	<0.01	<0.01	3.10	0.156

For all variables, the variance inflation factor was <2.

Abbreviations as in Tables 2 and 3.

Chronic infarct regions of adversely remodeled left ventricle had higher MD (1.82 ± 0.10 vs 1.63 ± 0.11 × 10^−3^ mm^2^/s; *P <* 0.001), lower FA (0.23 ± 0.02 vs 0.28 ± 0.04; *P <* 0.001), lower E2A (36 ± 5° vs 48 ± 9°; *P <* 0.001), lower RHM (6% ± 3% vs 17% ± 6%), and higher LHM (27% ± 11% vs 18% ± 9%; *P <* 0.001) than infarcted regions of nonadversely remodeled left ventricle, as well as higher native T1 (1,472 ± 117 ms vs 1,340 ± 95 ms; *P <* 0.001) and ECV (60% ± 12% vs 48% ± 15%; *P <* 0.001). Between the early and 12-month scans, infarct MD serially decreased in nonadverse remodelers from 1.71 ± 0.12 to 1.63 ± 0.11 × 10^−3^ mm^2^/s (*P <* 0.001), but it serially increased in adverse remodelers from 1.78 ± 0.13 to 1.82 ± 0.10 × 10^−3^ mm^2^/s (*P <* 0.001). There was a positive correlation between the serial changes in LVEDVi and MD, native T1, and ECV (Supplemental Figures 4A to 4C) of infarct regions over 12 months. There were no significant serial changes in FA, E2A, or RHM in infarct regions.

Chronic adjacent regions of adverse remodelers had higher MD (1.54 ± 0.05 vs 1.49 ± 0.06 × 10^-3^ mm^2^/s; *P <* 0.001), higher native T1 (1,264 ± 46 ms vs 1,214 ± 47 ms; *P <* 0.001), and higher ECV (29% ± 3% vs 27% ± 3%; *P <* 0.001) in comparison with adjacent regions of non-remodelers (Supplemental Table 2). Similarly, remote myocardium of adverse remodelers had higher MD (1.51 ± 0.06 vs 1.46 ± 0.06 × 10^-3^ mm^2^/s; *P =* 0.004), higher native T1 (1,270 ± 57 ms vs 1,189 ± 55 ms; *P <* 0.001), and higher ECV (31% ± 5% vs 27% ± 4%; *P <* 0.001) than the remote myocardium of nonadverse remodelers (Supplemental Table 2). Correlations between native T1, ECV, and MD of adjacent and remote regions and LVEDVi are shown in Supplemental Figures 4D to 4I). The other DT-CMR parameters did not directly correlate with serial change in LVEDVi in adjacent or remote regions.

### DT-CMR predictors of 12-month LVEF

**Figure 5:**
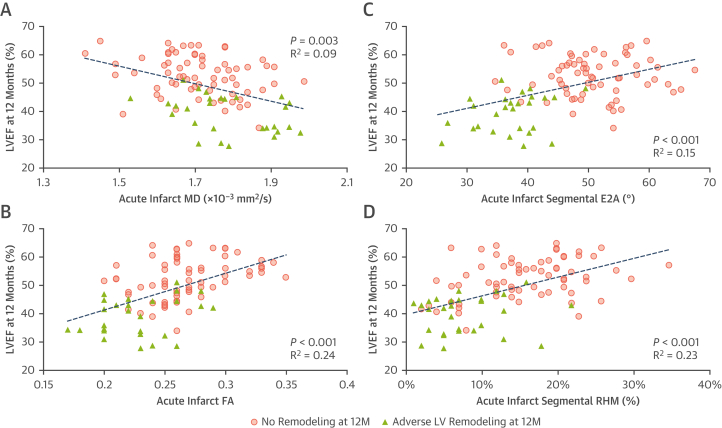
Relationship Between Tissue Characteristics of Chronic Infarct, Adjacent, and Remote Regions With Serial Change in LV Size **(A)** Increased MD, **(B)** decreased FA, **(C)** decreased absolute E2A, and **(D)** decreased proportions of RHM in acutely infarcted myocardium were all associated with lower LVEF at 12 months following STEMI. Abbreviations as in Figures 1 to 3.

As well as looking at adverse remodeling, univariate linear regression analysis was also carried out to identify acute predictors of long-term LVEF. Several CMR-based characteristics of the early scan were found to be significantly associated with LVEF at 12 months (Table 3, Figure 5); among DT-CMR parameters, these included infarct MD (r^2^ = 0.091), infarct FA (r^2^ = 0.244), infarct E2A (r^2^ = 0.151), and infarct RHM (r^2^ = 0.233). Following multivariate linear regression analysis adjusting for factors including infarct size and ECV, baseline LVEF (β = 0.352; *P =* 0.001), baseline LVEDVi (β = −0.212; *P =* 0.014), infarct FA (β = 0.246; *P =* 0.008), and infarct RHM (β = 0.177; *P =* 0.049) were independently associated with LVEF at 12 months. Acute infarct E2A of <45° had 85% sensitivity and 73% specificity, and RHM <12% had 85% sensitivity and 68% specificity for predicting LVEF of <35% at 12 months (Figure 4B).

## Discussion

To the best of our knowledge, this is the first study to report and compare the predictive relevance of early DT-CMR biomarkers against previously established CMR parameters for long-term adverse remodeling following STEMI. Our results also provide novel mechanistic insight into the short- and long-term pathophysiological sequelae of STEMI. Our main findings are as follows:

1. In the acute stages following STEMI, the diffusion of water molecules in infarcted myocardium of patients who undergo adverse remodeling by 12 months is more isotropic. The myocardial sheetlets in the infarct region are unable to adopt their usual orientation in systole, as suggested by low absolute E2A values, and they have less RHM, thereby highlighting the early loss of organization among subendocardial myocytes.
2. Over the 12 months post-STEMI, diffusion becomes more unrestricted in the infarcted myocardium of adversely remodeled hearts.
3. Reduced sheetlet angularity (E2A) and increased cardiomyocyte disorganization (FA) in acutely infarcted myocardium are independent predictors of adverse remodeling at 12 months. Acute infarct FA is also independently associated with LVEF at 12 months.

These findings can help elucidate some of the pathophysiologic mechanisms that drive adverse remodeling following STEMI and provide novel biomarkers for risk stratification.

### Impact of microstructural changes post-STEMI on LV geometry

The pathophysiology of myocardial ischemia is known to be linked to transmural variance in vascular compliance, thus making subendocardial myocytes the most vulnerable.^17^ Although the degree of infarct transmurality is known to be closely associated with LV remodeling^1^ the mechanisms linking infarct extent with change in LV shape are poorly understood. Histologic studies suggest that, following infarction, degradation of intermyocyte collagen struts leads to slippage between muscle bundles that results in wall thinning and impaired contractility.^2^ Connective tissue then infiltrates the myocyte compartment in an attempt to unite disrupted myocytes and improve scar tissue integrity. To resist LV dilatation, the replacement scar tissue requires sufficient tensile strength to overcome the stretching forces from increased preload.^1^

Through our results, we show that preservation of the sheetlet angularity during systole (higher E2A) and organization of cardiomyocyte arrangement (higher FA), particularly among subendocardial myocytes (higher proportion of RHM) in the acutely infarcted zones, play crucial roles in maintaining LV geometry and function.

MD reflects the diffusivity of water molecules. In healthy specimens, cell membranes provide biophysiologic barriers for diffusion. Meanwhile, the diffusion of water molecules is more unrestricted in areas of edema or in extracellular space because of the lack of cell membranes. Myocardial edema is expected to resolve over 12 months post-STEMI, but both MD and ECV serially increased in infarct regions of adverse remodelers over 12 months, thus alluding to an expansion in extracellular space.^7^^,^^8^ This serial increase directly correlated with the degree of LV dilatation. From this finding, we can propose that scar tissue with increased extracellular space (increased MD) and extensive cardiomyocyte disorganization (low FA) likely lacks the tensile strength needed to resist the stretching forces from increased preload post-STEMI. Chronic infarct segments of adverse remodelers also retained significantly lower E2A (reflecting the loss of sheetlet angularity during systole) and RHM (loss of organization among subendocardial cardiomyocytes) than non-remodelers, a finding demonstrating that the acute changes in axons of microstructural components remain fixed over time. Without histologic corroboration, it remains uncertain whether the significantly higher LHM in adverse remodelers amounts from a relative reduction in cardiomyocytes in RHM or an actual increase in the subepicardial cardiomyocytes.

The impact of adverse remodeling is known to extend beyond the infarct zones; the acute increase in preload detected by mechanoreceptors stimulates cellular hypertrophy and up-regulation of contractile assembly units in noninfarcted myocardium.^1^ Carrick et al^13^ previously observed that left ventricles that went on to remodel following STEMI had significantly higher native T1 in their remote myocardium during their early scans and attributed this feature to edema. In our cohort, even at 12 months, adverse remodelers had significantly higher T1, ECV, and MD in their remote and adjacent regions than nonadverse remodelers. In the convalescent phase (ie, in the absence of edema), this again likely reflects a maladaptive expansion in extracellular space, the exact cause of which warrants further studies.

### Added value of DT-CMR

Infarct size and MVO detected using LGE are already recognized as excellent prognostic biomarkers following MI. LGE provides accurate measurement of chronic infarct size but, with the exception of MVO detection, does not provide quantitative estimation of the severity of injury to underlying fibers and sheetlets. This finding is especially relevant in the acute stages following MI, where factors such as edema add complexity to LGE interpretation. Native T1, T2, and ECV mapping can provide quantification of edema and fibrosis by assessing tissue composition; however, this composition undergoes dynamic change immediately post-MI, thus making it challenging to differentiate between infarcted and edematous myocardium at this stage.^18^ Unlike conventional techniques, DT-CMR characterizes myocardial microstructure by inferring the orientations of microstructural components, something previously possible only in postmortem examinations. Hence DT-CMR is well suited for detecting irreversible injury. Our results suggest that changes in the axes of microstructural components are detectable within 1 week post-MI and persist over 12 months. This finding likely explains why parameters such as FA and E2A were independently associated with adverse remodeling even after accounting for clinical and angiographic factors. DT-CMR imaging can therefore complement current clinical and imaging risk factors in early prognostic risk-stratification following MI, thereby prompting earlier initiation of aggressive heart failure treatment and device therapy to patients at highest risk of adverse outcomes.

### Study limitations

Conclusions drawn from this study are based on correlations with published evidence and other CMR imaging biomarkers, whereas validation with histologic specimens would be preferable. Acquisitions of additional sequences such as T2 mapping would have been preferable but were omitted to reduce scan times. By allowing for noncontrast and free-breathing acquisitions, spin echo DT-CMR offers some practical benefits in the context of acute imaging post-STEMI; however, acquisition was limited to only 3 slices, and technical developments are needed to allow full LV coverage in shorter scan times. Acquisitions were also acquired at a single time point in the cardiac cycle (peak-systole), whereas LGE and mapping acquisitions were acquired in diastole. Postprocessing can be labor intensive, and clinical implementation requires further optimization, particularly with tractography postprocessing for accurate definition of HA variation across the myocardium and scar borders.

## Conclusions

Following MI, extensive cardiomyocyte disorganization as evidenced by low FA, acute loss of sheetlet angularity as evidenced by low E2A, and a greater loss of organization among RHM corresponding to subendocardium are all associated with long-term adverse remodeling and functional impairment. Longitudinal changes in MD suggest that diffusion becomes more unrestricted globally in adversely remodeled hearts, thus highlighting diffuse interstitial changes in infarcted, adjacent, and remote myocardium. In comparison with other more standardized CMR techniques, DT-CMR parameters from early scans were independently associated with adverse LV remodeling at 12 months. These findings warrant further validation in larger, multicenter DT-CMR studies.

Perspectives **COMPETENCY IN MEDICAL KNOWLEDGE:** There is a need for early imaging biomarkers to identify patients at high risk of adverse LV remodeling post-MI. DT-CMR has the potential to meet this need because it allows for noninvasive, noncontrast assessment of acute, irreversible changes in myocardial architecture in vivo. In this study of 100 patients with STEMI, we demonstrated that patients are at significantly greater risk of adverse LV remodeling post-MI if their acutely infarcted myocardium exhibits reduced FA (signifying isotropic diffusion from cardiomyocyte disorganization) and reduced E2A (signifying that the underlying myocardial sheetlets remain in a hypoangulated state during systole).

**TRANSLATIONAL OUTLOOK:** In this study, serial CMR scans were undertaken on patients with STEMI to establish potential associations between DT-CMR and long-term adverse LV remodeling, as well as to elucidate underlying pathophysiologic mechanisms. DT-CMR provides quantitative information on the histologic state of the myocardium that can complement clinical and imaging risk factors, including LVEF and LGE in prognostic stratification post-MI. Conclusions drawn from this study are based on correlations with published evidence and other more validated CMR parameters, whereas validation with histologic specimens would be preferable. Our findings warrant further validation in larger, multicenter DT CMR studies.

## Funding Support and Author Disclosures

Dr Das is a PhD student at the University of Leeds and has received funding from Heart Research UK (RG2668/18/20). Dr Stoeck has received funding from the Swiss National Science Foundation grant PZ00P2_174144. Dr Plein has received funding from a British Heart Foundation (BHF) Chair (CH/16/2/32089). Dr Dall’Armellina has received funding from a BHF Intermediate Clinical Research Fellowship (FS/13/71/30378). All other authors have reported that they have no relationships relevant to the contents of this paper to disclose.

## References

[1] St John Sutton M., Lee D., Rouleau J.L. (2003). Left ventricular remodeling and ventricular arrhythmias after myocardial infarction. Circulation.

[2] Pfeffer M.A., Braunwald E. (1990). Ventricular remodeling after myocardial infarction. Experimental observations and clinical implications. Circulation.

[3] Nielles-Vallespin S., Khalique Z., Ferreira P.F. (2017). Assessment of myocardial microstructural dynamics by in vivo diffusion tensor cardiac magnetic resonance. J Am Coll Cardiol.

[4] Das A., Kelly C., Teh I. (2021). Acute microstructural changes after ST-segment elevation myocardial infarction assessed with diffusion tensor imaging. Radiology.

[5] Scollan D.F., Holmes A., Winslow R., Forder J. (1998). Histological validation of myocardial microstructure obtained from diffusion tensor magnetic resonance imaging. Am J Physiol.

[6] Moulin K., Viallon M., Romero W. (2020). MRI of reperfused acute myocardial infarction edema: ADC quantification versus T1 and T2 mapping. Radiology.

[7] Wu R., An D.A., Hu J. (2018). The apparent diffusion coefficient is strongly correlated with extracellular volume, a measure of myocardial fibrosis, and subclinical cardiomyopathy in patients with systemic lupus erythematosus. Acta Radiol.

[8] Das A., Chowdhary A., Kelly C. (2021). Insight into myocardial microstructure of athletes and hypertrophic cardiomyopathy patients using diffusion tensor imaging. J Magn Reason Imaging.

[9] Kung G.L., Vaseghi M., Gahm J.K. (2018). Microstructural infarct border zone remodeling in the post-infarct swine heart measured by diffusion tensor MRI. Front Physiol.

[10] Abdullah O.M., Drakos S.G., Diakos N.A. (2014). Characterization of diffuse fibrosis in the failing human heart via diffusion tensor imaging and quantitative histological validation NIH public access. NMR Biomed.

[11] Fihn S.D., Blankenship J.C., Alexander K.P. (2014). 2014 ACC/AHA/AATS/PCNA/SCAI/STS focused update of the guideline for the diagnosis and management of patients with stable ischemic heart disease: a report of the American College of Cardiology/American Heart Association Task Force on Practice Guidelines, and the American Association for Thoracic Surgery, Preventive Cardiovascular Nurses Association, Society for Cardiovascular Angiography and Interventions, and Society of Thoracic Surgeons. J Am Coll Cardiol.

[12] Alam M.H., Auger D., McGill L.A. (2016). Comparison of 3 T and 1.5 T for T2∗ magnetic resonance of tissue iron. J Cardiovasc Magn Reson.

[13] Carrick D., Haig C., Rauhalammi S. (2016). Prognostic significance of infarct core pathology revealed by quantitative non-contrast in comparison with contrast cardiac magnetic resonance imaging in reperfused ST-elevation myocardial infarction survivors. Eur Heart J.

[14] Messroghli D.R., Moon J.C., Ferreira V.M. (2017). Clinical recommendations for cardiovascular magnetic resonance mapping of T1, T2, T2 and extracellular volume: a consensus statement by the Society for Cardiovascular Magnetic Resonance (SCMR) endorsed by the European Association for Cardiovascular Imaging. J Cardiovasc Magn Reson.

[15] Cerqueira M.D., Weissman N.J., Dilsizian V. (2002). Standardized myocardial segmentation and nomenclature for tomographic imaging of the heart. Circulation.

[16] DeLong E.R., DeLong D.M., Clarke-Pearson D.L. (1988). Comparing the areas under two or more correlated receiver operating characteristic curves: a nonparametric approach. Biometrics.

[17] Algranati D., Kassab G.S., Lanir Y. (2011). Why is the subendocardium more vulnerable to ischemia? A new paradigm. Am J Physiol Heart Circ Physiol.

[18] Kidambi A., Motwani M., Uddin A. (2017). Myocardial extracellular volume estimation by CMR predicts functional recovery following acute MI. J Am Coll Cardiol Img.

