# Supplementary material for: Pathophysiology of LV Remodeling Following STEMI: A Longitudinal Diffusion Tensor CMR Study
Source: JACC Cardiovasc Imaging. 2023 Feb;16(2):159–71. doi: 10.1016/j.jcmg.2022.04.002 (PMC9902278; doi:10.1016/j.jcmg.2022.04.002)
Supplement: Supplemental Data [file mmc1.docx]

# Supplemental Methods

## Pulse sequence parameters and CMR protocol

CMR examinations were performed on a 3.0 Tesla ﻿Philips Achieva TX system (Philips, Best, The Netherlands) equipped with a 32-channel cardiac phased array receiver coil, MultiTransmit technology and high-performance gradients with Gmax = 80mT/m and slew rate = 100 mT/m/ms. ﻿﻿A full blood count, including haematocrit was measured at the time of intravenous cannulation. Cine-imaging used a balanced steady-state free precession (bSSFP) pulse sequence (echo time (TE)/repetition time (TR)/flip angle 1.3ms/2.6ms/40°, spatial resolution 1.6×2.0×10mm, typical temporal resolution 25ms). MOLLI T1 mapping parameters were as follows: 5/3/0 acquisition, TE/TR 2.1/0.82 msec, flip angle = 20°, spatial resolution 0.91x0.91x8mm, SENSE 2 acceleration, cardiac delay time 777ms. T2* mapping used a gradient echo sequence (TE 1.18 ms, TR 17 ms, flip angle 20°, spatial resolution 1.8x1.8x8mm). Repeat MOLLI T1 mapping was performed at 15 minutes post-contrast (4/3/2 acquisition, TE/TR 2.1/0.82 msec, flip angle 35°, spatial resolution 0.91×0.91×8mm, SENSE 2 acceleration, cardiac delay time 728ms), and LGE imaging at 16-20 minutes post contrast (inversion recovery-prepared T1-weighted gradient echo, inversion time according to Look-Locker scout, spatial resolution 0.91×0.91×8mm, TR/TE/flip angle 3.7/2.0/25°). In addition to the LGE short-axis slices, a contiguous stack of LGE images was acquired immediately afterwards. To ensure consistent slice positioning and infarct analysis between time points, image acquisition were performed in 3 matching short-axis positions by acquiring the central 3 slices of 5 parallel short-axis slices spaced equally from the mitral annulus to the LV apical cap. ﻿ Additional short axis LGE images were acquired in mid-systole for direct comparison in order to assist contouring of DT-CMR maps.

## CMR Analysis

## On LGE images, the threshold used for identifying infarcted tissue was set to 5 standard deviations above remote myocardial tissue signal intensity. Computer assisted planimetry was used to derive total infarct size (expressed as percentage of LV mass) as well as segmental infarct transmurality (percentage of enhancement for each of the 16 segments of the AHA model). MVO was defined as dark zones within an area of hyperenhancement on LGE images at 15-mins post-contrast administration.

## DT-CMR post processing

Post-processing was performed by an investigator blinded to clinical data, using custom-built software developed in house using MATLAB (MathWorks, MA, USA). All diffusion images were co-registered via a mutual-information-based, multi-resolution, affine scheme using the elastix toolbox. After data rejection, 10 ± 2 DWI repetitions were available per diffusion gradient orientation for tensor reconstruction (inclusive of base and mid slices only; apical data was excluded from the study due to persistent data quality issues, typically resulting from unsuppressed fat, localised signal loss or low signal-noise ratio). Based on the registered data, magnitude averaged images were generated according to diffusion direction and b-value; diffusion tensors were calculated using a linear least-squares approach. ROIs were deemed appropriate for MD and FA where the normal characterisation does not show significant spatial inhomogeneity in the healthy population. Both HA and E2A demonstrate a higher degree of spatial inhomogeneity in the normal population, in particular HA where we observe the transmural variation in tissue characterisation; as a result, ROI-based values would be incapable of capturing accurately these local changes and segmental quantification using the AHA model was employed instead.

ROI analysis

Regions of interest (ROI) were manually planimetered for the analysis of MD, FA, native T1, T2 and ECV on corresponding maps. As recommended by the standards set by the European Association for Cardiovascular Imaging, ROIs were drawn on greyscale images to avoid bias. Very small ROIs (<20 pixels) were avoided. ROIs were sampled away from endo- and epicardial borders to avoid the effects of partial voluming. For each patient in each affected slice, three ROIs were drawn corresponding to: infarct (positive for LGE), adjacent oedematous myocardium (negative LGE, raised native T1 [departmental threshold >1240ms]) and remote myocardium (opposite to the infarct) – as shown in **Supplemental Figure 1**. Infarct and remote ROIs were identified on the LGE image and copied across to the other maps. Adjacent ROIs were identified on native T1 maps and copied to the other maps.

For cases with intramyocardial haemorrhage (IMH), in order to avoid the paramagnetic susceptibility effects of iron, care was taken to avoid sampling ROIs from areas of MVO (as seen on LGE) and IMH (identified using T2* mapping), as shown in **Supplemental Figure 2**. Infarct myocardium was identified on LGE and areas of IMH was identified on T2* maps. ROIs were drawn and copied across to parametric and DT-CMR maps. Infarct ROIs on parametric and DT-CMR maps were adjusted so they did not sample areas of IMH. For each patient, the location of ROIs from acute scan were used as a visual reference for sampling ROIs from 12-month scans, so that sampling occurred from near-identical locations.

Intra-subject Reproducibility

In order to assess the test-retest reproducibility of our sequence, DT-CMR was acquired on 5 healthy controls using the methods described in the manuscript. To ensure consistent slice positioning, scans were acquired back-to-back in the same session. The global results including MD, FA, E2A and HA are provided in **Supplemental Table 1**.

MD was found to be the most reproducible parameter with a coefficient of variation (CoV) of 2.68, closely followed by FA, E2A, proportions of RHM, CM and LHM respectively. Bland-Altman plots for intrasubject reproducibility are shown in **Supplemental Figure 3.**

Comparison of case with and without MVO

MD and FA maps were analysed using ROI approach as detailed above, specifically avoiding areas of MVO when present. Unlike MD and FA, E2A and HA values varies transmurally across the myocardium in healthy subjects. Due to this expected spatial inhomogeneity, it is challenging to define the ‘normal range’ for a specific region within the myocardium, making ROI analysis unfeasible, hence segmental analysis was performed for E2A and HA maps. In our results, patients with MVO had lower E2A (40±7° vs 49±3°, p<0.001) and lower RHM (9±7% vs 16±8%, p<0.001) in their infarct segments, as shown in **Supplemental Table 2**.

As it was not feasible to perform ROI analysis and exclude the areas of MVO on E2A and HA maps, we instead performed separate analysis, excluding all patients with MVO (n=28). In the remaining 72 cases, patients who went on to adversely remodel still had significantly lower segmental E2A (38±3° vs 51±37°, p<0.001) and lower proportions of RHM (9±6% vs 17±8%, p<0.001) in their infarct segments, as shown below in **Supplemental Table 3**.

Results from the follow up scans at 12 months are shown in **Supplemental Table 4**.

**Supplemental Table 1: Intra-subject reproducibility results**

|  | **Bias** | **Limits of agreement** | | **Coefficient of variation (%)** |
| --- | --- | --- | --- | --- |
|  |  | **Lower** | **Upper** |  |
| MD (x10^-3^mm^2^/s) | -0.02 | -0.13 | 0.10 | 2.68 |
| FA | -0.02 | -0.09 | 0.05 | 6.82 |
| E2A (°) | 0.87 | -7.97 | 9.71 | 6.92 |
| HA proportions | |  |  |  |
| RHM (%) | <0.01 | -0.06 | 0.06 | 6.92 |
| CM (%) | <0.01 | -0.14 | 0.15 | 5.99 |
| LHM (%) | -0.01 | -0.10 | 0.08 | 19.70 |

**Supplemental Table 2: Comparison of DT-CMR parameters in patients with and without MVO.**

**Supplemental Table 3: Comparison of DT-CMR parameters in patients without MVO (n=72)**

**Supplemental Table 4: Follow-up CMR results at 12-months**

| **CMR Findings** | **Follow up scan at 12 months** | | | |
| --- | --- | --- | --- | --- |
|  | **All patients**  **(n=100)** | **No remodelling at 12 months (n=68)** | **Adverse remodelling at 12 months (n=32)** | **P Value** |
| Left ventricular ejection fraction (%) | 49±9 | 53±7 | 41±9 | <0.001 |
| BSA Indexed LV End-diastolic volume (ml/m^2^) | 85±22 | 78±16 | 103±24 | <0.001 |
| Left ventricular mass (g) | 106±25 | 103±21 | 112±33 | 0.107 |
| Infarct Size (% of left ventricle mass) | 13±10 | 9±7 | 27±22 | <0.001 |
| Infarct transmurality (%) | 52±32 | 10±5 | 65±20 | <0.001 |
| Microvascular obstruction size (g) | - | - | - | - |
| Wall thickness of infarct segment (mm) | 6.4±1.5 | 6.8±1.4 | 6.1±1.8 | 0.024 |
| **Remote regions:** |  |  |  |  |
| Native T1 (ms) | 1214±67 | 1189±55 | 1270±57 | <0.001 |
| Extracellular Volume (%) | 28±4 | 27±4 | 31±5 | <0.001 |
| T2 (ms) | 45±9 | 44±10 | 49±6 | 0.060 |
| Mean Diffusivity (x10^-3^mm^2^/s) | 1.48±0.06 | 1.46±0.06 | 1.51±0.06 | 0.004 |
| Fractional Anisotropy | 0.34±0.03 | 0.35±0.04 | 0.34±0.04 | 0.318 |
| Absolute secondary eigenvector angle (°)* | 48±7 | 49±6 | 46±7 | 0.022 |
| Right-handed myocyte orientation (%)* | 21±8 | 20±8 | 23±9 | 0.268 |
| Circumferential myocyte orientation (%)* | 68±11 | 69±12 | 65±12 | 0.166 |
| Left-handed myocyte orientation (%)* | 11±8 | 11±9 | 12±7 | 0.464 |
| **Adjacent regions:** |  |  |  |  |
| Native T1 (ms) | 1230±52 | 1214±47 | 1264±46 | <0.001 |
| Extracellular Volume (%) | 28±3 | 27±3 | 29±3 | <0.001 |
| T2 (ms) | 49±5 | 48±4 | 50±5 | 0.111 |
| Mean Diffusivity (x10^-3^mm^2^/s) | 1.50±0.06 | 1.49±0.06 | 1.54±0.05 | <0.001** |
| Fractional Anisotropy | 0.34±0.03 | 0.34±0.03 | 0.33±0.03 | 0.054 |
| Absolute secondary eigenvector angle (°)* | 46±8 | 48±8 | 45±7 | 0.192 |
| Right-handed myocyte orientation (%)* | 17±8 | 18±9 | 16±9 | 0.420 |
| Circumferential myocyte orientation (%)* | 67±13 | 76±13 | 74±14 | 0.348 |
| Left-handed myocyte orientation (%)* | 6±9 | 6±14 | 10±10 | 0.272 |
| **Infarct regions:** |  |  |  |  |
| Native T1 (ms) | 1380±124 | 1340±95 | 1472±117 | <0.001 |
| Extracellular Volume (%) | 52±17 | 48±15 | 60±12 | <0.001 |
| T2 (ms) | 50±13 | 51±7 | 54±7 | 0.029 |
| Presence of IMH on T2* mapping (n,%) | - | - | - | - |
| Mean Diffusivity (x10^-3^mm^2^/s) | 1.70±0.14 | 1.63±0.11 | 1.82±0.10 | <0.001** |
| Fractional Anisotropy | 0.26±0.04 | 0.28±0.04 | 0.23±0.02 | <0.001 |
| Absolute secondary eigenvector angle (°)* | 44±10 | 48±9 | 36±5 | <0.001 |
| Right-handed myocyte orientation (%)* | 14±8 | 17±6 | 6±3 | <0.001 |
| Circumferential myocyte orientation (%)* | 68±10 | 68±10 | 67±12 | 0.830 |
| Left-handed myocyte orientation (%)* | 18±9 | 15±8 | 27±11 | <0.001 |

**Supplemental Figure 1**

*ROI analysis of remote, adjacent and infarct segments. Please note ROIs were originally drawn on a greyscale image to avoid bias; colour scales have been added to this figure for the reader’s benefit.*

**Supplemental Figure 2**

*ROI analysis for cases with intramyocardial haemorrhage. Please note ROIs were originally drawn on a greyscale image to avoid bias; colour scales have been added to this figure for the reader’s benefit.*

Supplemental Figure 3: Bland-Altman plots for intra-subject reproducibility


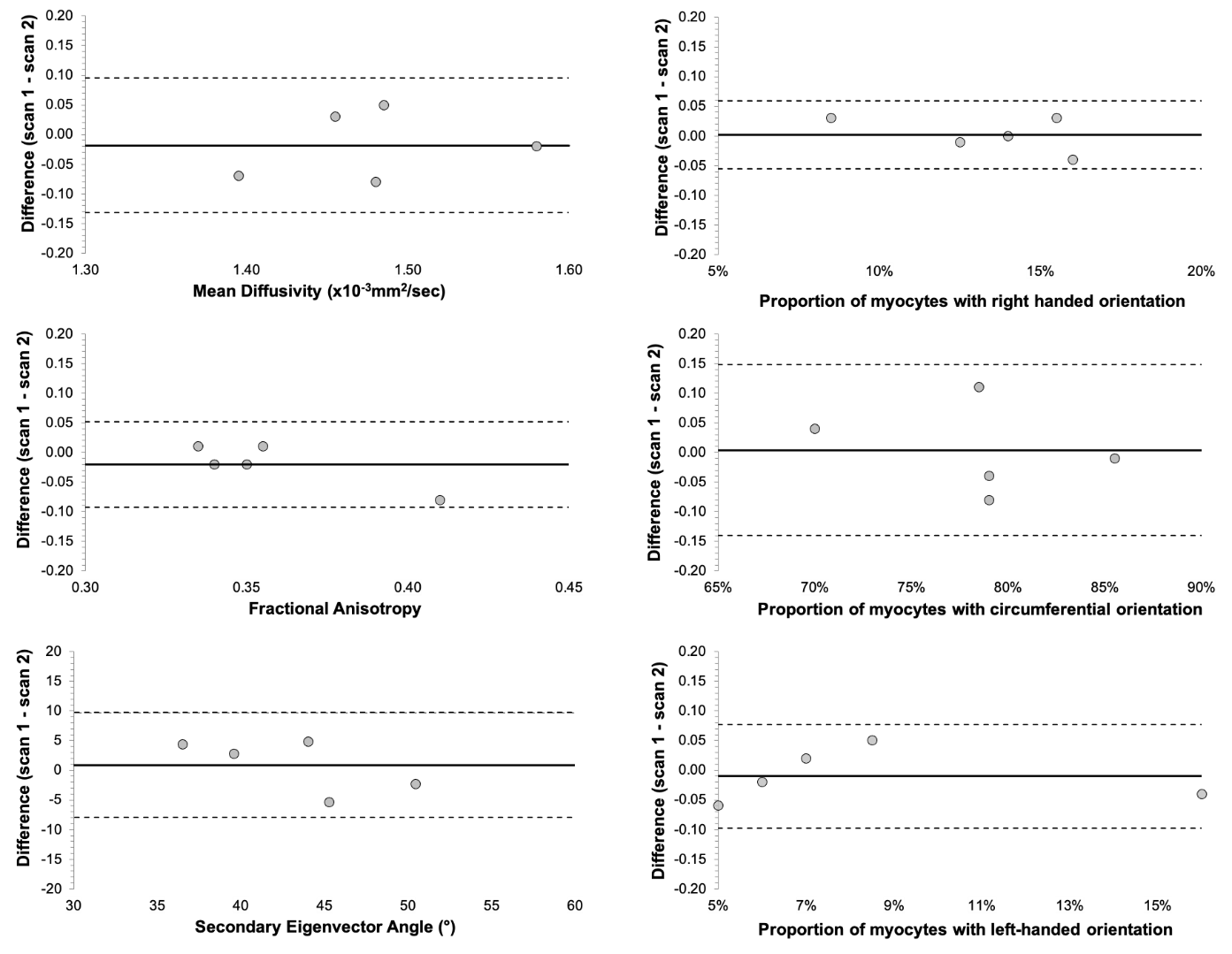


*Bland-Altman plots for repeat measurements in 5 healthy controls, showing the difference between scan 1 and scan 2 for different DT-CMR parameters. The central (thick) line represents the bias and the dashed lines represent the 95% limits of agreement.*

**Supplemental Figure 4: Correlations between acute DT-CMR parameters and LVEF % at 12-months**

*Serial changes in mean diffusivity (MD, panel A), native T1 (panel B) and extracellular volume (ECV, panel C) of infarct regions correlated with serial change in indexed left ventricular end-diastolic volume (LVEDVi). The MD, native T1 and ECV of adjacent (panels D, E, F) and remote (panels G, H, I) regions also correlate with change in LVEDVi over 12-months.*
